# The genome sequence of the white ermine, *Spilosoma lubricipeda *Linnaeus 1758

**DOI:** 10.12688/wellcomeopenres.17190.1

**Published:** 2021-10-14

**Authors:** Douglas Boyes, Peter W.H. Holland, University of Oxford and Wytham Woods Genome Acquisition Lab, Darwin Tree of Life Barcoding collective, Wellcome Sanger Institute Tree of Life programme, Wellcome Sanger Institute Scientific Operations: DNA Pipelines collective, Tree of Life Core Informatics collective, Darwin Tree of Life Consortium

**Affiliations:** 1 UK Centre for Ecology & Hydrology, Wallingford, OX10 8BB, UK; 2 Department of Zoology, University of Oxford, Oxford, OX1 3SZ, UK

**Keywords:** Spilosoma lubricipeda, white ermine, genome sequence, chromosomal

## Abstract

We present a genome assembly from an individual male *Spilosoma lubricipeda *(the white ermine; Arthropoda; Insecta; Lepidoptera; Erebidae). The genome sequence is 587 megabases in span. The majority of the assembly is scaffolded into 30 chromosomal pseudomolecules, with the Z sex chromosome assembled.

## Species taxonomy

Eukaryota; Metazoa; Ecdysozoa; Arthropoda; Hexapoda; Insecta; Pterygota; Neoptera; Endopterygota; Lepidoptera; Glossata; Ditrysia; Noctuoidea; Erebidae; Arctiinae; Spilosoma; *Spilosoma lubricipeda* Linnaeus 1758 (NCBI:txid875880).

## Introduction

*Spilosoma lubricipeda* (White ermine) is found across much of Eurasia but has decreased in abundance significantly in the UK in recent decades, with the cause(s) being unknown ( Fox *et al*., 2013) ( Prescott *et al*., 2019) The genome of *S. lubricipeda* was sequenced as part of the Darwin Tree of Life Project, a collaborative effort to sequence all of the named eukaryotic species in the Atlantic Archipelago of Britain and Ireland. Here we present a chromosomally complete genome sequence for *S. lubricipeda*, based on one male specimen from Wytham Woods, Oxfordshire, UK.

## Genome sequence report

**Figure 1.**
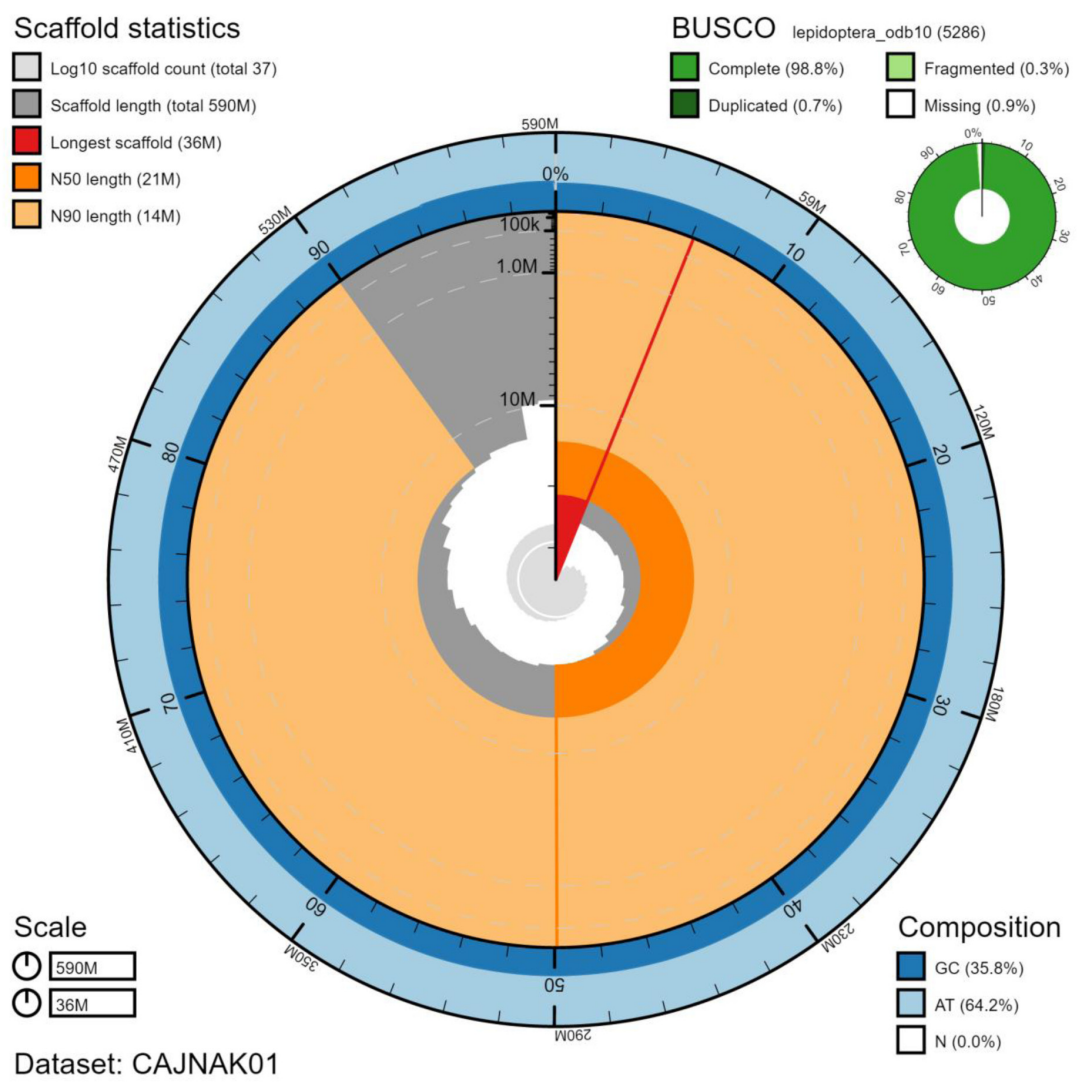
Genome assembly of *Spilosoma lubricipeda*, ilSpiLubr1.1: metrics. The BlobToolKit Snailplot shows N50 metrics and BUSCO gene completeness. An interactive version of this figure is available at https://blobtoolkit.genomehubs.org/view/ilSpiLubr1.1/dataset/CAJNAK01/snail.

The genome was sequenced from a single male *S. lubricipeda* collected from Wytham Woods, Oxfordshire, UK (latitude 51.768, longitude -1.337). A total of 39-fold coverage in Pacific Biosciences single-molecule long reads and 43-fold coverage in 10X Genomics read clouds were generated. Primary assembly contigs were scaffolded with chromosome conformation Hi-C data. Manual assembly curation corrected 22 missing/misjoins and removed 2 haplotypic duplications, reducing the assembly length by 0.09% and increasing the scaffold number by 3.70%. The final assembly has a total length of 587 Mb in 37 sequence scaffolds with a scaffold N50 of 21 Mb ( Table 1). Of the assembly sequence, 99.98% was assigned to 30 chromosomal-level scaffolds, representing 29 autosomes (numbered by sequence length), and the Z sex chromosome ( Figure 1– Figure 4; Table 2). The assembly has a BUSCO ( Simão *et al*., 2015) v5.1.2 completeness of 98.8% using the lepidoptera_odb10 reference set. While not fully phased, the assembly deposited is of one haplotype. Contigs corresponding to the second haplotype have also been deposited.

**Table 1.** Genome data for *Spilosoma lubricipeda*, ilSpiLubr1.1.

*Project accession data*	
Assembly identifier	ilSpiLubr1.1
Species	*Spilosoma lubricipeda*
Specimen	ilSpiLubr1
NCBI taxonomy ID	NCBI:txid875880
BioProject	PRJEB42957
BioSample ID	SAMEA7520525
Isolate information	Male, abdomen
*Raw data accessions*	
PacificBiosciences SEQUEL II	ERR6406203
10X Genomics Illumina	ERR6054439-ERR6054442
Hi-C Illumina	ERR6054438
*Genome assembly*	
Assembly accession	GCA_905220595.1
*Accession of alternate haplotype*	GCA_905220605.1
Span (Mb)	587
Number of contigs	57
Contig N50 length (Mb)	21
Number of scaffolds	37
Scaffold N50 length (Mb)	21
Longest scaffold (Mb)	25
BUSCO * genome score	C:98.8%[S:98.1%,D:0.7%],F :0.3%,M:0.9%,n:5286

*BUSCO scores based on the lepidoptera_odb10 BUSCO set using v5.1.2. C= complete [S= single copy, D=duplicated], F=fragmented, M=missing, n=number of orthologues in comparison. A full set of BUSCO scores is available at https://blobtoolkit.genomehubs.org/view/ilSpiLubr1.1/dataset/CAJNAK01/busco.

**Figure 2.**
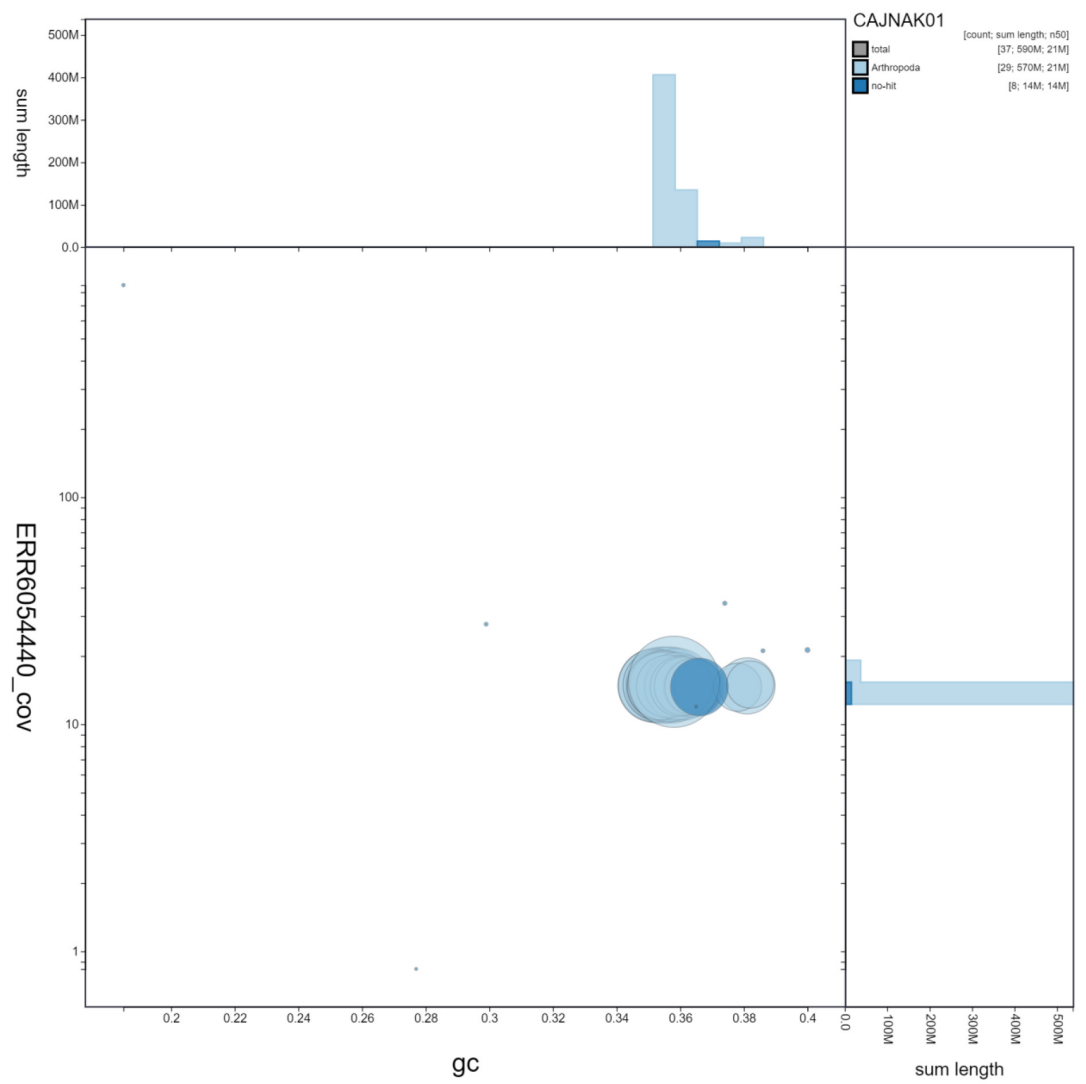
Genome assembly of *Spilosoma lubricipeda*, ilSpiLubr1.1: GC coverage. BlobToolKit GC-coverage plot. Scaffolds are coloured by phylum. Circles are sized in proportion to scaffold length. Histograms show the distribution of scaffold length sum along each axis. An interactive version of this figure is available at https://blobtoolkit.genomehubs.org/view/ilSpiLubr1.1/dataset/CAJNAK01/blob.

**Figure 3.**
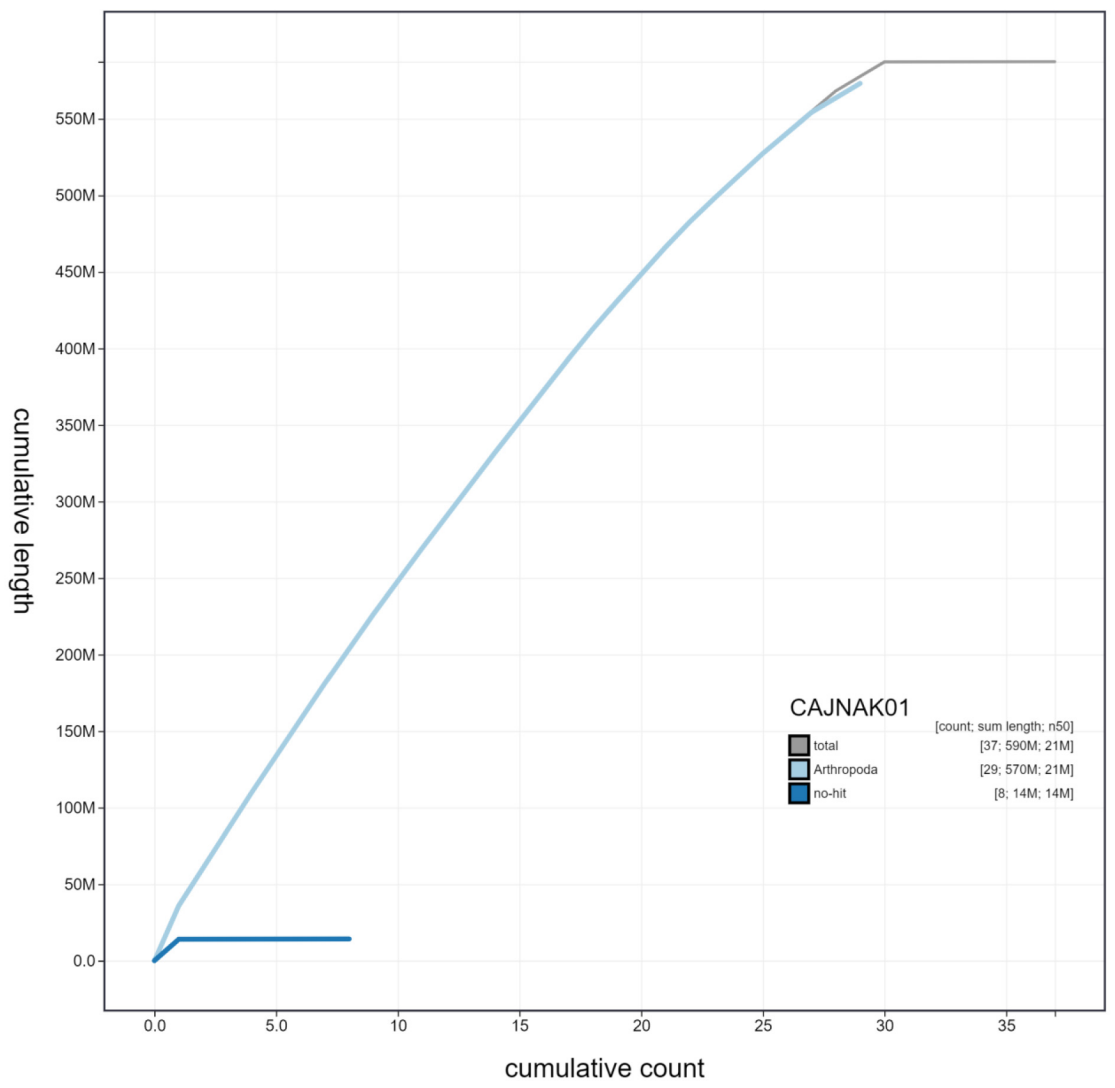
Genome assembly of *Spilosoma lubricipeda*, ilSpiLubr1.1: cumulative sequence. BlobToolKit cumulative sequence plot. The grey line shows cumulative length for all chromosomes. Coloured lines show cumulative lengths of chromosomes assigned to each phylum using the buscogenes taxrule. An interactive version of this figure is available at https://blobtoolkit.genomehubs.org/view/ilSpiLubr1.1/dataset/CAJNAK01/cumulative.

**Figure 4.**
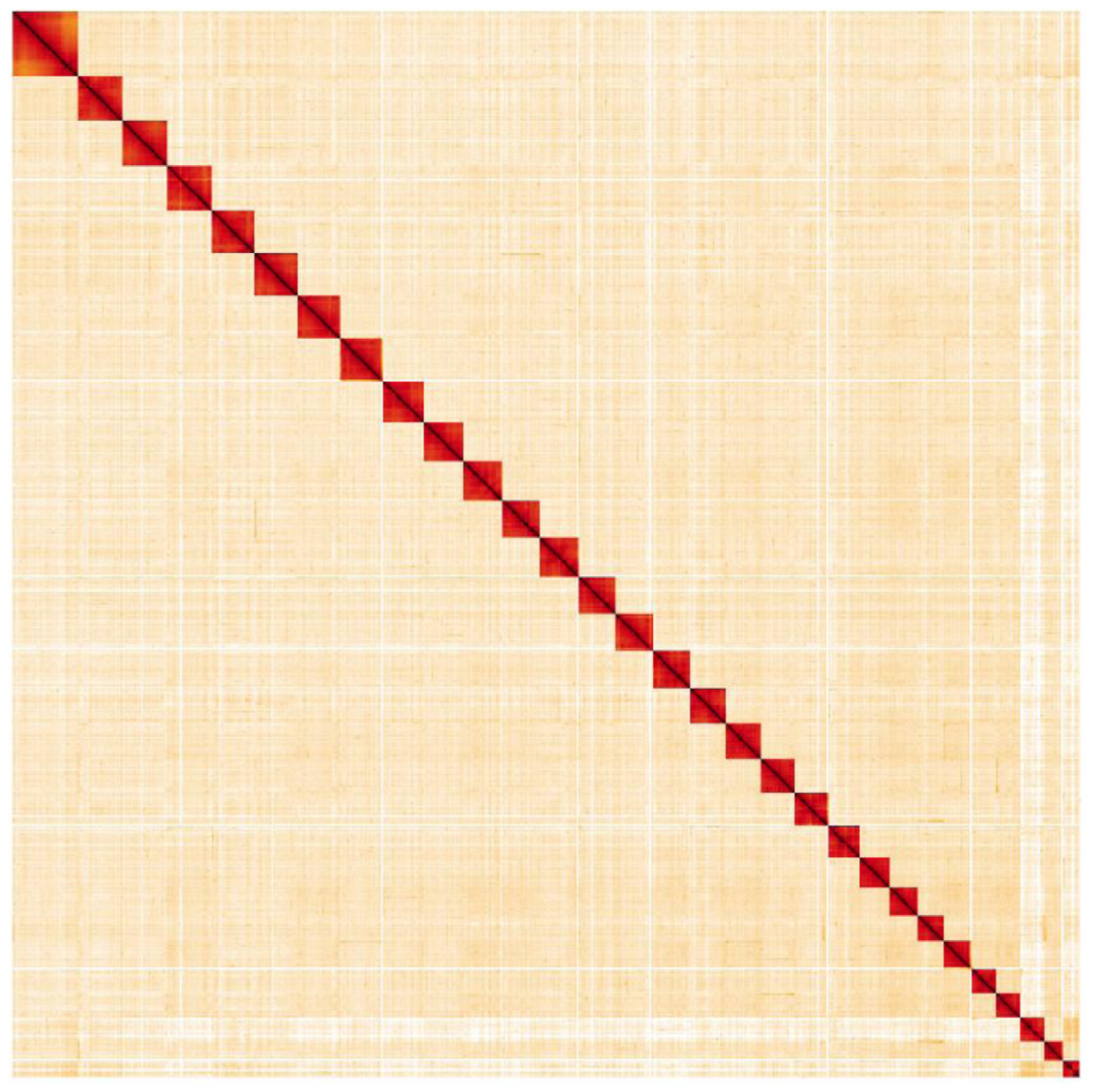
Genome assembly of *Spilosoma lubricipeda*, ilSpiLubr1.1: Hi-C contact map. Hi-C contact map of the ilSpiLubr1.1 assembly, visualised in HiGlass.

**Table 2.** Chromosomal pseudomolecules in the genome assembly of *Spilosoma lubricipeda*, ilSpiLubr1.1.

INSDC accession	Chromosome	Size (Mb)	GC%
HG992275.1	1	24.84	35.7
HG992276.1	2	24.64	35.5
HG992277.1	3	24.48	35.4
HG992278.1	4	23.97	35.4
HG992279.1	5	23.77	35.6
HG992280.1	6	23.46	35.2
HG992281.1	7	22.82	35.9
HG992282.1	8	22.61	35.2
HG992283.1	9	21.71	35.4
HG992284.1	10	21.21	35.5
HG992285.1	11	21.05	35.3
HG992286.1	12	21.03	35.5
HG992287.1	13	20.64	35.7
HG992288.1	14	20.45	35.6
HG992289.1	15	20.26	35.4
HG992290.1	16	20.07	35.5
HG992291.1	17	19.45	35.4
HG992292.1	18	18.38	35.9
HG992293.1	19	17.77	36.1
HG992294.1	20	17.67	35.9
HG992295.1	21	16.57	35.6
HG992296.1	22	15.29	36
HG992297.1	23	14.91	36.4
HG992298.1	24	14.47	36.1
HG992299.1	25	14.02	36.6
HG992300.1	26	13.41	36.4
HG992301.1	27	13.33	38.1
HG992302.1	28	9.66	37.8
HG992303.1	29	9.33	38.2
HG992274.1	Z	35.93	35.8
HG992304.1	MT	0.02	18.6
-	Unplaced	0.15	36.7

## Methods

A single male *S. lubricipeda* was collected from Wytham Woods, Oxfordshire, UK (latitude 51.768, longitude -1.337) by Douglas Boyes, University of Oxford using a light trap. The specimens were snap-frozen in dry ice using a CoolRack before transferring to the Wellcome Sanger Institute (WSI).

DNA was extracted at the Tree of Life laboratory, WSI. The ilSpiLubr1 sample was weighed and dissected on dry ice with tissue set aside for Hi-C sequencing. Abdomen tissue was disrupted to a fine powder using a Biomasher tissue homogeniser. Fragment size analysis of 0.01-0.5 ng of DNA was then performed using an Agilent FemtoPulse. High molecular weight (HMW) DNA was extracted using the Qiagen MagAttract HMW DNA extraction kit. Low molecular weight DNA was removed from a 200-ng aliquot of extracted DNA using 0.8X AMpure XP purification kit prior to 10X Chromium sequencing; a minimum of 50 ng DNA was submitted for 10X sequencing. HMW DNA was sheared into an average fragment size between 12-20 kb in a Megaruptor 3 system with speed setting 30. Sheared DNA was purified by solid-phase reversible immobilisation using AMPure PB beads with a 1.8X ratio of beads to sample to remove the shorter fragments and concentrate the DNA sample. The concentration of the sheared and purified DNA was assessed using a Nanodrop spectrophotometer and Qubit Fluorometer and Qubit dsDNA High Sensitivity Assay kit. Fragment size distribution was evaluated by running the sample on the FemtoPulse system.

Pacific Biosciences HiFi circular consensus and 10X Genomics read cloud sequencing libraries were constructed according to the manufacturers’ instructions. Sequencing was performed by the Scientific Operations core at the Wellcome Sanger Institute on Pacific Biosciences SEQUEL II and Illumina HiSeq X instruments. Hi-C data were generated from abdomen tissue using the Arima v2.0 kit and sequenced on HiSeq X.

Assembly was carried out with Hifiasm ( Cheng *et al*., 2021); haplotypic duplication was identified and removed with purge_dups ( Guan *et al*., 2020). The assembly was polished with the 10X Genomics Illumina data by aligning to the assembly with longranger align, calling variants with freebayes ( Garrison & Marth, 2012). One round of the Illumina polishing was applied. Scaffolding with Hi-C data ( Rao *et al*., 2014) was carried out with SALSA2 ( Ghurye *et al*., 2019). The assembly was checked for contamination and corrected using the gEVAL system ( Chow *et al*., 2016) as described previously ( Howe *et al*., 2021). Manual curation was performed using gEVAL, HiGlass and Pretext. The mitochondrial genome was assembled using MitoHiFi ( Uliano-Silva *et al*., 2021). The genome was analysed and BUSCO scores generated within the BlobToolKit environment ( Challis *et al*., 2020). Table 3 contains a list of all software tool versions used, where appropriate.

**Table 3.** Software tools used.

Software tool	Version	Source
Hifiasm	0.1.2	Cheng *et al*., 2021
purge_dups	1.2.3	Guan *et al*., 2020
longranger	2.2.2	https://support.10xgenomics.com/genome-exome/ software/pipelines/latest/advanced/other-pipelines
freebayes	1.3.1-17-gaa2ace8	Garrison & Marth, 2012
MitoHiFi	1.0	Uliano-Silva *et al*., 2021
SALSA2	2.2	Ghurye *et al*., 2019
gEVAL	N/A	Chow *et al*., 2016
HiGlass	1.11.6	Kerpedjiev *et al*., 2018
PretextView	0.1.x	https://github.com/wtsi-hpag/PretextView
BlobToolKit	2.6.2	Challis *et al*., 2020

The materials that have contributed to this genome note have been supplied by a Darwin Tree of Life Partner. The submission of materials by a Darwin Tree of Life Partner is subject to the Darwin Tree of Life Project Sampling Code of Practice. By agreeing with and signing up to the Sampling Code of Practice, the Darwin Tree of Life Partner agrees they will meet the legal and ethical requirements and standards set out within this document in respect of all samples acquired for, and supplied to, the Darwin Tree of Life Project. Each transfer of samples is further undertaken according to a Research Collaboration Agreement or Material Transfer Agreement entered into by the Darwin Tree of Life Partner, Genome Research Limited (operating as the Wellcome Sanger Institute), and in some circumstances other Darwin Tree of Life collaborators.

## Data availability

European Nucleotide Archive: Spilosoma lubricipeda (white ermine). Accession number PRJEB42957: https://identifiers.org/ena.embl:PRJEB42957

The genome sequence is released openly for reuse. The *S. lubricipeda* genome sequencing initiative is part of the Darwin Tree of Life (DToL) project. All raw sequence data and the assembly have been deposited in INSDC databases.The genome will be annotated and presented through the Ensembl pipeline at the European Bioinformatics Institute. Raw data and assembly accession identifiers are reported in Table 1.
