# Supplementary material for: The genome sequence of the white ermine, Spilosoma lubricipeda Linnaeus 1758
Source: Wellcome Open Res. 2021 Oct 14;6:271. [Version 1] doi: 10.12688/wellcomeopenres.17190.1 (PMC8567691; doi:10.12688/wellcomeopenres.17190.1)
Supplement: Peer review history

# Peer review: Reviewer response for version 1

**DOI:** 10.21956/wellcomeopenres.18993.r46444

**Published:** 2021-11-04

**Authors:** Paul Frandsen

**Affiliations:** 1 Department of Plant and Wildlife Sciences, Brigham Young University, Provo, UT, USA

This is a fantastic genome created by a fantastic project. I love these papers because they emerge from a group that has clearly thought a lot about their methods and pipeline, which can inform the rest of us! I have a couple of minor comments:

 1. Is the collection record shared somewhere (GPS coordinates, etc.)? If not, it would be good to include. I couldn't find it on ENA, but it's possible this is due to my own unfamiliarity with the ENA.

 2. The paper mentions that the PacBio HiFi library was prepared according to manufacturer instructions, but the exact protocol used was missing (standard protocol/low input protocol/ultra-low protocol).

 3. For the polishing step were all Chromium reads mapped to the haploid or the diploid assembly? In my own work, I've noticed that in areas of substantial heterozygosity, short reads often do a poor job of polishing due to inaccurate mapping. In my experience, at least, this is partly alleviated by using a diploid genome assembly or by attempting to phase reads before the polishing step.

Are sufficient details of methods and materials provided to allow replication by others?

Partly

Is the rationale for creating the dataset(s) clearly described?

Yes

Are the datasets clearly presented in a useable and accessible format?

Yes

Are the protocols appropriate and is the work technically sound?

Partly

Reviewer Expertise:

Insect genomics

I confirm that I have read this submission and believe that I have an appropriate level of expertise to confirm that it is of an acceptable scientific standard.

# Peer review: Reviewer response for version 1

**DOI:** 10.21956/wellcomeopenres.18993.r46441

**Published:** 2021-11-03

**Authors:** Petr Nguyen

**Affiliations:** 1 University of South Bohemia, Ceske Budejovice, Czech Republic

The authors report a chromosome-level assembly of the white ermine, *Spilosoma lubricipeda*, sequenced by PacBio and 10x Genomics technologies. Decrease of *S. lubricipeda* abundance in the UK is mentioned in the introduction but it is not clear how it is relevant for genome sequencing. It should be elaborated. 

 The *S. lubricipeda* assembly is 587 Mb long. Although the *S. lubricipeda *genome size is not known, some comparison with a genome size inferred from k-mers would be nice. Anyway, the assembly length seems to be close to a genome size of its congener, *S. virginica* (626 Mb according to www.genomesize.com). 99.98% of sequence was assigned to 30 chromosome-level scaffolds, which is interesting as Robinson (1971, Lepidoptera genetics) ^1^ provides chromosome number n=31 for this species citing two independent references. The Z chromosome is surprisingly large in *S. lubricipeda. *The karyotype n=31 is ancestral in Lepidoptera and comparison with genome of some other species with n=31 such as *Plutella xylostella*, *Spodoptera litura*, or *Melitaea cinxia* could reveal fusion between the Z chromosome and an autosome which occurred in the sampled *S. lubricipeda *population. The authors should address the above mentioned in their genome sequence report.

 The method section describes the pipeline but not the parameters used. For better reproducibility, parameters used for individual processing and assembly steps should be specified.

Are sufficient details of methods and materials provided to allow replication by others?

Partly

Is the rationale for creating the dataset(s) clearly described?

Partly

Are the datasets clearly presented in a useable and accessible format?

Yes

Are the protocols appropriate and is the work technically sound?

Yes

Reviewer Expertise:

Genetics of Lepidoptera

I confirm that I have read this submission and believe that I have an appropriate level of expertise to confirm that it is of an acceptable scientific standard, however I have significant reservations, as outlined above.

## References

[1] : Lepidoptera Genetics: International Series of Monographs in Pure and Applied Biology: Zoology. *Pergamon Press* .1971;46: 10.1016/C2013-0-01588-5 10.1016/C2013-0-01588-5

# Peer review: Reviewer response for version 1

**DOI:** 10.21956/wellcomeopenres.18993.r46443

**Published:** 2021-11-02

**Authors:** Dominik R. Laetsch

**Affiliations:** 1 Institute of Evolutionary Biology, University of Edinburgh, Edinburgh, UK

This article describes the sampling, sequencing, assembly, scaffolding, curation, and quality control that led to the *Spilosoma lubricipeda *genome assembly. The steps are described in sufficient detail to allow replication and are consistent with current best practices. Relevant data has been deposited on INSDC databases and is accessible.

 Minor comments:

- Richard Fox in the comments points out that the reference Prescott *et al*., 2019 should be edited. Since he is one of the authors of that book he is probably right.
- The same sentence (first sentence in introduction) is also missing a full stop after the citations.
- The introduction could be expanded a bit to include more information about why the genome is of interest to the research community.

Are sufficient details of methods and materials provided to allow replication by others?

Yes

Is the rationale for creating the dataset(s) clearly described?

Yes

Are the datasets clearly presented in a useable and accessible format?

Yes

Are the protocols appropriate and is the work technically sound?

Yes

Reviewer Expertise:

Evolutionary Biology, Bioinformatics, Genomics

I confirm that I have read this submission and believe that I have an appropriate level of expertise to confirm that it is of an acceptable scientific standard.

# Peer review: Reviewer response for version 1

**DOI:** 10.21956/wellcomeopenres.18993.r46442

**Published:** 2021-10-28

**Authors:** Merly Escalona

**Affiliations:** 1 Department of Biomolecular Engineering, University of California–Santa Cruz, Santa Cruz, CA, USA

The paper reports a chromosome level assembly of the White ermine moth. The quality of the genome assembly seems impressive at first sight and corresponds to an excellent resource for the scientific community. However, there are some things missing from the report that are important for a type of data note/genome announcement paper that is presented here. 

- First, seems necessary and in line with published genome notes from the same journal to have a picture of the species. 
- There are metrics other than BUSCO scores that complement the quality assessment of a genome assembly, i.e. k-mer completeness and per base quality (QV).
- While there’s information related to the amount of read coverage generated for Pacific Biosciences (PacBio) HiFi reads and 10X Chromium data, there’s no information regarding the coverage for the Arima HiC libraries. It might not be common to describe ‘coverage’ for HiC data but knowing the number of reads generated makes the description of the sequencing data generated consistent.
- Related to the mitochondrial genome assembly: MitoHiFi looks for a closely related species and uses it to guide the assembly for the species at hand. It is then relevant to know which species was used.
- Description of the software version used is not consistent throughout the paper. At the Genome sequence report section, you identify the version of the BUSCO score/program with v5.1.2 but then the rest of the software tools do not have the version next to them but on Table 3. 
- There are missing citations in the text. Methods section, 4th paragraph. HiGlass and Pretext.
- Also, in the Methods section, 4th paragraph, on the sentence before last:
  “The genome was analyzed and BUSCO scores generated within the BlobToolKit environment ( Challis et al., 2020).” - Although I understand the context of the sentence, the flow of the paragraphs seems to indicate that this sentence refers to the mitochondrial assembly which is incorrect. 

Are sufficient details of methods and materials provided to allow replication by others?

No

Is the rationale for creating the dataset(s) clearly described?

Yes

Are the datasets clearly presented in a useable and accessible format?

Yes

Are the protocols appropriate and is the work technically sound?

Yes

Reviewer Expertise:

Genome assembly

I confirm that I have read this submission and believe that I have an appropriate level of expertise to confirm that it is of an acceptable scientific standard, however I have significant reservations, as outlined above.
